# From siege to spillover: how Gaza’s health crisis threatens global infection spread

**DOI:** 10.1093/ije/dyag072

**Published:** 2026-05-20

**Authors:** Muna Abed Alah, Peter Valentine Coyle

**Affiliations:** Clinical Effectiveness Department, Primary Health Care Corporation, P.O. Box 26555, Doha, Qatar; Virology Division, Department of Laboratory Medicine and Pathology, Hamad Medical Corporation, P.O. Box 3050, Doha, Qatar; Department of Biomedical Science, College of Health Sciences, QU Health, Qatar University, P.O. Box 2713, Doha, Qatar

## Introduction

Viral transmission within communities is shaped by a complex interaction of environmental, social, host, and pathogen-related factors, including population density, contact patterns, mobility, and host susceptibility [1]. It is further optimized by the introduction or emergence of new variants in conditions of overcrowding in which the affected population lacks or has suboptimal immunity to the variant [1]. Armed conflict, population displacement, and the collapse of essential health services create environments in which viral pathogens and new variants can spread unchecked and undergo accelerated evolution [2]. Historical precedents highlight the profound impact of such conditions on the emergence and spread of infectious diseases. The 1918 influenza pandemic, often referred to as the Spanish flu, serves as a stark example. The movement of troops during World War I, coupled with overcrowded military camps and inadequate sanitation, facilitated the rapid transmission of the 1918 H1N1 influenza virus [3, 4]. The conflict provided conditions for widespread infection with the new reassorted virus and its later variants that drove subsequent waves [5].

In more recent history, the Democratic Republic of Congo (DRC) has experienced multiple Ebola outbreaks, often exacerbated by ongoing armed conflicts. The 2018–20 outbreak in the DRC was particularly severe, with >3000 cases and >2000 deaths [6]. The conflict in the region hindered response efforts, delayed the establishment of treatment centers, and contributed to the spread of the virus through disrupted healthcare services and population displacement [6, 7].

The ongoing humanitarian crisis in Gaza exemplifies such a scenario [8–10]. With >2 million people living under siege conditions, overcrowding, nonfunctioning sanitation, lack of clean water, disrupted vaccination campaigns, and widespread malnutrition converge to create a perfect storm for large-scale outbreaks of infectious diseases [11].

While conflict conditions facilitate the transmission of a broad spectrum of pathogens including bacteria, parasites, and deoxyribonucleic acid (DNA) viruses, this paper focuses primarily on ribonucleic acid (RNA) viruses because they possess the greatest intrinsic evolutionary potential. Apart from human coronaviruses, RNA viruses lack proofreading during genomic replication, producing high mutation rates and extensive genetic diversity that enable immune evasion, ecological disruption, and host population changes [12]. Their short generation times, capacity for recombination and reassortment, and ability to persist in immunologically weakened or immune-naive hosts make them particularly vulnerable to the selective pressures generated by overcrowding, displacement, malnutrition, and health-system collapse [13]. In parallel, recent studies have demonstrated that humanitarian evacuations and cross-border patient transfers from conflict settings such as Gaza can facilitate the international spread of multidrug-resistant bacteria, with the documented transmission of resistant organisms across national borders following medical evacuation and displacement, showing that bacterial infections also pose transnational risks in humanitarian crises [14]. However, because the mechanisms and speed of evolutionary change are most pronounced in RNA viruses, the present analysis concentrates on their potential to generate variants capable of regional or global spillover.

### Mechanisms of viral evolution

Viral evolution is driven by multiple processes that interact at both the molecular and population levels. At the core is the high error rate of RNA-dependent polymerases, coupled with a lack of proofreading, which generates frequent “point mutations” during replication [12, 13]. These mutations create a swarm of closely related genomes, known as a “quasispecies,” in which diversity allows rapid adaptation to selective pressures such as host immunity, antivirals, or ecological change [13]. Prolonged infections, particularly in immunocompromised or malnourished hosts, expand quasispecies diversity and increase the chance of advantageous variants emerging [15, 16].

Beyond point mutations, viruses also exchange larger units of genetic material. “Reassortment” occurs in viruses with segmented genomes, such as influenza and rotavirus, when coinfection allows genome segments from different parental strains to be packaged together, yielding progeny with novel segment combinations [17, 18]. “Recombination,” in contrast, involves template switching during replication within the same genome segment, producing mosaic sequences; this mechanism is prominent in enteroviruses and coronaviruses [19]. Both processes can create abrupt genomic change with major consequences for host range, immune evasion, and transmissibility.

These molecular mechanisms form the basis of broader population-level phenomena. “Antigenic drift” refers to the gradual accumulation of mutations in genes encoding the viral surface proteins, steadily eroding immune recognition and necessitating periodic vaccine updates, as seen in influenza [20]. “Antigenic shift,” by contrast, is the sudden appearance of new antigenic profiles, typically through reassortment, which has driven past influenza pandemics [4, 21]. In influenza A, antigenic shift denotes the abrupt emergence of viral strains with substantially altered surface antigens, typically hemagglutinin (HA) and/or neuraminidase (NA), resulting from reassortment between human and avian (or other animal) strains. Such shifts can generate novel influenza A subtypes against which the human population has limited or no preexisting immunity and have historically precipitated influenza pandemics. By contrast, reassortment in rotaviruses can generate new combinations of the two major outer capsid proteins VP7 (G type, glycoprotein) and VP4 (P type, protease-sensitive protein), which define the virus’s serotype. These events lead to genotype shifts or strain replacement, altering the prevalence and distribution of circulating G/P combinations and potentially affecting vaccine effectiveness [22]. Together, quasispecies dynamics, reassortment, recombination, drift, and shift constitute the evolutionary engine that enables viral emergence and persistence.

### Environmental drivers of viral evolution in conflict settings

The conflict-driven collapse of health systems, combined with war-related environmental factors, amplifies viral evolutionary processes, as summarized in Table 1. “Overcrowded shelters,” “mass displacement,” and “poor sanitation” facilitate intense transmission and viral replication, raising the likelihood of coinfections that drive reassortment and recombination, as documented for enteroviruses [19, 23, 24]. “Disrupted immunization” campaigns reduce herd immunity, prolonging the circulation of vaccine-preventable diseases, including vaccine-derived polioviruses for which oral polio vaccine is used [25, 26]. In addition, “the destruction of healthcare infrastructure and limited access to diagnostics and treatment” further exacerbate these risks [26, 27]. Delayed detection of infections and shortages of antivirals or supportive care prolong viral replication, especially in immunocompromised or malnourished hosts, increasing opportunities for mutation, recombination, and reassortment [2]. “The inability to conduct effective surveillance” allows emerging variants to circulate unchecked, creating conditions for rapid viral adaptation, immune escape, and potential spillover beyond the conflict zone.

**Table 1:** Conflict-driven environmental drivers of viral evolution.

Driver	How it arises in conflict settings	Contribution to viral adaptation/spillover risk
Overcrowding and mass displacement	Refugee camps, shelters, urban crowding	Increased coinfections and transmission rates facilitate the exchange of genetic material and accumulation of mutations, generating novel variants
Malnutrition/food insecurity	Limited access to adequate protein and essential micronutrients due to restricted humanitarian aid, coupled with the destruction of agricultural land and crops	Prolonged infections in malnourished hosts increase viral replication cycles, facilitating mutations and the emergence of adaptive variants
Disrupted vaccination/low herd immunity	Collapse of routine immunization programs, compounded by shortages of vaccines and healthcare personnel, and limited access to populations due to military restrictions	Reduced population immunity allows circulating viruses to persist, accumulate mutations, and reassort, potentially producing immune-escape variants
Poor sanitation/contaminated water	Damaged water and sewage infrastructure	Increases fecal–oral viral transmission, promotes coinfections, genetic diversification, and circulation of vaccine-derived strains
Healthcare system collapse (delayed diagnosis, limited access to treatment and diagnostics)	Hospitals and laboratories destroyed, shortages of antivirals, limited diagnostic capacity	Delayed detection and prolonged untreated infections lead to higher viral loads and extended replication cycles, increasing opportunities for mutations, recombination, and reassortment, and allowing emerging variants to circulate unchecked

“Malnutrition” further amplifies viral evolution. Deficiencies in protein and micronutrients impair immune responses, prolong infections, and increase viral replication, creating conditions for adaptive mutations [15, 16]. Experimental models on mice show that deficiencies in nutrients such as selenium or vitamin E can drive the emergence of more virulent influenza and coxsackievirus strains, suggesting a mechanism through which nutritional crises may contribute to viral adaptation and pathogenicity [28, 29]. Historical precedents, including hypotheses proposed regarding the 1918 influenza pandemic, suggest that wartime conditions, including malnourished troops, troop mobilization, and unsanitary encampments, may have influenced the apparent virulence and facilitated the spread of the newly emerged 1918 influenza H1N1 virus [30].

### Case example: Gaza’s humanitarian crisis and viral evolution

The current humanitarian crisis in Gaza provides a striking example of how conflict conditions create a crucible for viral evolution. Since October 2023, >1.9 million people (>85% of the population) have been displaced into overcrowded shelters, with families living in spaces of <2 m^2^ per person in certain shelters [31]. Access to clean water has dramatically declined, falling well below the World Health Organization (WHO) emergency minimum, while the majority of water and sanitation infrastructure has been rendered nonfunctional [32]. This collapse has led to massive outbreaks of enteric infections with >577 000 cases of acute watery diarrhea and 995 000 cases of acute respiratory infections reported as of August 2024 [33]. Before the escalation in hostilities, an average of 2000 cases of diarrhea in children <5 years old were recorded per month throughout 2021 and 2022 [34]. Between 2023 and 2024, acute watery diarrhea cases rose by 36-fold (412 840 cases vs 11 562 cases), bloody diarrhea by 24-fold (7504 cases vs 311 cases), and acute jaundice syndrome (hepatitis A) by 384-fold, with children <5 years old representing 65% of the acute watery diarrhea cases in 2025 [35]. Prior to 7 October 2023, acute respiratory infections in Gaza were monitored through functioning surveillance and primary healthcare systems, and generally followed expected seasonal patterns. In 2022, ∼82 000 cases of COVID-19 were reported in Gaza, resulting in >400 deaths [36]. Following the escalation of hostilities, WHO surveillance documented a marked rise in respiratory infections, with >50 000 cases reported within weeks, particularly among displaced populations living in overcrowded conditions [34].

Prior to 7 October 2023, routine immunization coverage in the Gaza Strip was very high, with overall childhood vaccination rates of ∼98% and polio vaccine coverage (third dose) estimated at 89% in 2023 [37, 38]. Following the escalation of hostilities, the health system suffered extensive damage and routine vaccination coverage declined to <70%, with many immunization facilities no longer operational [39]. Routine immunization programs have been severely disrupted, resulting in incomplete coverage for multiple vaccine-preventable diseases, including polio and measles [25]. In January 2024, circulating vaccine-derived poliovirus type 2 (cVDPV2) was detected in both sewage samples and in a confirmed paralytic case after 25 years of being polio-free, illustrating how gaps in immunization can allow the spread of vaccine-derived poliovirus [25]. In response to the polio outbreak, an emergency vaccination campaign was launched, though not all planned rounds could be completed. Following the ceasefire, integrated catch-up immunization efforts have been launched to restore routine vaccination coverage for children who missed essential vaccines, including rotavirus, pneumococcal conjugate, and measles-containing vaccines [39].

The conflict in Gaza has led to the widespread destruction of healthcare infrastructure, severely hindering access to essential medical services. As of May 2025, ∼94% of hospitals in Gaza have been damaged or destroyed, severely compromising the ability of the healthcare system to provide essential services [40]. This widespread destruction has rendered only 19 out of 36 hospitals operational, with many functioning at minimal capacity due to shortages of medical supplies and staff, and infrastructure damage [40].

The conflict has also led to the repurposing of several hospitals as shelters for displaced individuals, exacerbating overcrowding and increasing the risk of infectious-disease transmission [9, 41]. This situation has significantly hindered access to treatment, antivirals, and diagnostic services, further straining the already overwhelmed healthcare system. The collapse of healthcare facilities, coupled with restricted access to treatment and diagnostics, has significantly impaired the ability to manage and control infectious diseases, leading to increased morbidity and mortality among the population.

Compounding these risks, as of August 2025, over half a million people faced famine (Integrated Food Security Phase Classification—IPC Phase 5), with ≥20% of households unable to meet minimum food needs. Another 1.07 million are in Emergency (IPC Phase 4) and 396 000 in Crisis (IPC Phase 3) [42]. As mentioned earlier, malnutrition weakens host immunity and prolongs viral shedding, enabling intra-host viral evolution and increasing the likelihood of immune escape or enhanced transmissibility [16].

### Call to action

Taken together, these factors mirror historical settings in which novel viral variants have emerged, highlighting the urgent need for coordinated intervention. Immediate actions must include the restoration of water and sanitation infrastructure, reinforcement of routine immunization programs, provision of nutritional support, and expansion of genomic and environmental surveillance to detect emerging and reemerging pathogens.

Recent analyses suggest that the human toll of the Gaza conflict has been substantially underestimated [43]. While many deaths have resulted directly from violence, peer-reviewed and independent studies indicate that the overall mortality exceeds official counts, with civilians comprising the majority of fatalities in the most recent conflict [44]. The collapse of health services, combined with displacement, overcrowding, and food insecurity, is likely to have aggravated mortality through indirect pathways, including the spread of infectious diseases, thereby increasing both the death toll and the risk of regional spillover.

The widespread disruption of health services, infrastructure, and reporting capacity during the conflict has limited the functionality of the routine surveillance systems, prompting the WHO and partners to expand the use of the Early Warning, Alert and Response System (EWARS) to support timely outbreak detection and response in emergency conditions [45].

Equally critical is ensuring that healthcare facilities remain operational, displaced populations have safe access to care, and antiviral treatments and diagnostic tools are available. Achieving a sustained ceasefire and enabling the unrestricted entry of humanitarian aid are essential to implement these measures effectively. Delays could allow viral adaptation to accelerate, posing a risk not only to Gaza, but also to regional and global populations. Although population movement is currently restricted, limited pathways such as the movement of humanitarian workers, medical evacuations, and environmental or wastewater-associated pathogen spread could facilitate transmission to neighboring regions. Historical experience demonstrates that pandemics may be seeded by very small numbers of infected individuals crossing borders, with subsequent amplification determined by population susceptibility and public health conditions. A classic example is the 2003 SARS (severe acute respiratory syndrome) outbreak, in which a single symptomatic physician traveling from southern China spent one night in a Hong Kong hotel and transmitted the virus to multiple guests, who subsequently carried it to several countries, seeding an international outbreak [46]. The current ceasefire and gradual resumption of cross-border movement highlight the relevance of these pathways, reinforcing the need for vigilant surveillance and timely interventions. Protecting civilian health systems during conflict is both a moral imperative and a cornerstone of global health security.

## Data Availability

There are no new data associated with this article.
